# Evaluation of In Vitro Synergistic Effects of Tetracycline with Alkaloid-Related Compounds against Diarrhoeic Bacteria

**DOI:** 10.3390/ijms25116038

**Published:** 2024-05-30

**Authors:** Hayford Osei-Owusu, Johana Rondevaldova, Marketa Houdkova, Tomas Kudera, Tersia Needham, Anna Mascellani, Ladislav Kokoska

**Affiliations:** 1 Department of Crop Sciences and Agroforestry, Faculty of Tropical AgriSciences, Czech University of Life Sciences Prague, Kamycka 129, Suchdol, 16500 Prague, Czech Republic; osei-owusu@ftz.czu.cz (H.O.-O.); rondevaldova@ftz.czu.cz (J.R.); houdkovam@ftz.czu.cz (M.H.); kuderat@ftz.czu.cz (T.K.); 2 Department of Animal Science and Food Processing, Faculty of Tropical AgriSciences, Czech University of Life Sciences Prague, Kamycka 129, Suchdol, 16500 Prague, Czech Republic; needham@ftz.czu.cz; 3 Department of Food Science, Faculty of Agrobiology, Food and Natural Resources, Czech University of Life Sciences Prague, Kamycka 129, Suchdol, 16500 Prague, Czech Republic; mascellani@af.czu.cz

**Keywords:** antimicrobial agents, combination therapy, diarrhoea, antibacterial resistance, pathogens, synergistic effect

## Abstract

Diarrhoea remains an important public health concern, particularly in developing countries, and has become difficult to treat because of antibacterial resistance. The development of synergistic antimicrobial agents appears to be a promising alternative treatment against diarrhoeic infections. In this study, the combined effect of tetracycline together with either nitroxoline, sanguinarine, or zinc pyrithione (representing various classes of plant-based compounds) was evaluated in vitro against selected diarrhoeic bacteria (*Enterococcus faecalis*, *Escherichia coli*, *Listeria monocytogenes*, *Shigella flexneri*, *Vibrio parahaemolyticus*, and *Yersinia enterocolitica*). The chequerboard method in 96-well microtiter plates was used to determine the sum of the fractional inhibitory concentration indices (FICIs). Three independent experiments were performed per combination, each in triplicate. It was observed that the combination of tetracycline with either nitroxoline, sanguinarine, or zinc pyrithione produced synergistic effects against most of the pathogenic bacteria tested, with FICI values ranging from 0.086 to 0.5. Tetracycline–nitroxoline combinations produced the greatest synergistic action against *S. flexneri* at a FICI value of 0.086. The combinations of the agents tested in this study can thus be used for the development of new anti-diarrhoeic medications. However, studies focusing on their in vivo anti-diarrhoeic activity and safety are required before any consideration for utilization in human medicine.

## 1. Introduction

Diarrhoea is a worldwide problem [1] and a major cause of infant and childhood morbidity and mortality in developing countries [2]. Around 525,000 children under the age of five die from diarrhoea each year worldwide [3]. The most common causes of diarrhoea are parasites, viruses, and bacteria (e.g., *Escherichia coli*, *Listeria monocytogenes*, *Shigella* spp., *Vibrio* spp., and *Yersinia enterocolitica*) [4]. However, another bacterial pathogen such as *Enterococcus faecalis* may be a causative agent of diarrhoea, especially in elderly and immunocompromised patients [5,6,7]. Oral rehydration therapy is the mainstay of treatment for diarrhoea, including illnesses for which antibiotics are indicated, such as cholera and shigellosis [8,9]. For example, ciprofloxacin is a drug of choice for the treatment of *Shigella* dysentery [10] and doxycycline is the first-line treatment for *Vibrio cholerae* infection [11]. However, the extensive use of antibiotics disrupts the normal balance of the gut microbiota, which protects against infectious diarrhoea [12,13,14]. For instance, it has been reported that ceftriaxone, when ingested, can change the gut microbiota composition and cause diarrhoea in up to 50% of children [15]. Furthermore, the misuse of antibiotics can lead to the development of bacterial resistance, which is currently a threat to public health and safety, as it contributes to the difficulty of treating infections and results in increased mortality rates [16]. It has been reported that because of infections caused by multidrug-resistant bacteria, nearly 700,000 people die every year [17]. Under these circumstances, alternative antimicrobial agents and approaches are needed, such as the combined use of synergistic antimicrobial agents, to reduce the prevalence of diarrhoea whilst preventing the emergence of multidrug resistance in bacteria.

Combination therapy, based on the synergistic action of antimicrobial agents, is an emerging pharmacological strategy that is effective for the treatment of various infectious diseases. Antimicrobial combinations can be classified as synergistic when the interaction of two or more agents results in a combined effect that is greater than the sum of their activities when used individually. The use of synergistic antimicrobial combinations results in decreased therapeutical doses and/or minimum inhibitory concentrations (MICs) of the agents [18]. On the contrary, antagonism is a condition where one of the agents mitigates the action of the other and the combination is less effective than a single agent at the same concentration [19]. In recent times, the approach of utilizing synergism has drawn the attention of many researchers and pharmaceutical industries in an attempt to develop antibacterial agents with different mechanisms of actions that can decrease the required dosage of individual antimicrobials and broaden the spectrum of antimicrobial activity against diarrhoeic pathogens [20]. For example, a synergistic antimicrobial effect between tetracycline and amoxicillin has been observed against the diarrhoeic pathogens *Bacillus cereus*, *E. faecalis*, and *Salmonella typhi* [21]. The combination of β-lactams and inhibitors of β-lactamases, such as ampicillin with sulbactam (Unasyn, Pfizer, New York, NY, USA), is currently used to treat intra-abdominal infections caused by β-lactamase-producing strains of *Bacteroides fragilis*, *Enterobacter* spp., *E. coli*, and *Klebsiella* spp. [22]. The results of previous experiments suggest that combining conventional antibiotics with non-antibiotic agents, whose antibacterial effect is based on different mechanisms of action (e.g., plant alkaloids), can be an efficient approach for the discovery of drugs designed to combat diarrhoea. For example, the combination of tetracycline with berberine considerably reduced the volume, frequency, and duration of diarrhoea in patients in a randomized double-blind placebo clinical trial [23]. Another study reported the in vitro growth-inhibitory activity of sanguinarine in combination with streptomycin against a clinical isolate of a diarrhoea-causing *E. coli* [24]. Nevertheless, the anti-diarrhoeic potential of the combinations of antibiotics with alkaloid compounds has not yet been fully explored.

Alkaloids are organic compounds of natural origin which contain at least one nitrogen atom in their molecule [25,26]. Structural modifications of alkaloids have served as a basis for the development of many semisynthetic and synthetic drugs currently used in clinical practice [27]. Recently, the alkaloid sanguinarine and synthetic alkaloid analogues nitroxoline and zinc pyrithione have attracted the attention of researchers as compounds that are able to enhance the activity of antibiotics against diarrhoeic bacteria. Sanguinarine, a benzylisoquinoline alkaloid isolated from the plant *Sanguinaria canadensis* [28], is commonly used in mouthwash and toothpaste to treat and prevent gingivitis and related inflammatory conditions [29,30]. It has demonstrated a synergistic effect against diarrhoeic pathogens when combined with ciprofloxacin [31]. Similarly, nitroxoline is a drug used to treat infectious and inflammatory urogenital tract diseases [32] and has showed synergism when used in combination with ciprofloxacin against strains of diarrhoea-causing bacteria [31]. This agent is a derivative of 8-hydroxyquinoline, which is a compound previously detected in the plant *Centaurea diffusa* [33]. In addition, zinc pyrithione is a metal complex of pyrithione (a compound isolated from the plant *Polyalthia nemoralis*) [34] that is commonly used in cosmetic products to treat skin infections (e.g., dandruff) caused by *Malassezia* spp. [35,36]. Zinc pyrithione produces synergistic antibacterial effects when combined with ciprofloxacin [31]. However, studies aimed at identifying the possible interactions of nitroxoline, sanguinarine, and zinc pyrithione with other antibiotics are not currently known. Since tetracycline has shown synergistic antibacterial activities when used together with various other plant-derived agents [37,38], this study evaluates the combined effects of tetracycline with the above-mentioned alkaloid-related compounds against diarrhoea-causing bacteria, namely, *E. faecalis*, *E. coli*, *L. monocytogenes*, *Shigella flexneri*, *V. parahaemolyticus*, and *Y. enterocolitica* in vitro.

## 2. Results

When tetracycline was used in combination with either nitroxoline, sanguinarine, or zinc pyrithione, synergistic activity was observed against several diarrhoea-causing bacteria. The MIC values obtained for nitroxoline, sanguinarine, and zinc pyrithione against the different strains of bacteria tested ranged from 2 to 16, from 3.33 to 128, and from 8 to 16 µg/mL, respectively. According to the FICI values and the number of bacterial strains susceptible to the combination of agents tested, tetracycline produced the strongest interaction when combined with nitroxoline, followed by zinc pyrithione and sanguinarine.

Table 1 shows the individual and combined MICs of tetracycline and nitroxoline against the selected diarrhoea-causing bacteria, together with their corresponding fractional inhibitory concentration indices (FICIs). The tetracycline–nitroxoline combination produced various synergistic interactions against all bacterial strains, and the sum of the FICIs ranged from 0.086 to 0.5. The greatest synergistic effect was observed against *S. flexneri* (FICI: 0.086) at 0.063 µg/mL nitroxoline, resulting in a 16-fold decrease in tetracycline MIC (from 4 to 0.25 µg/mL). Additionally, the greatest MIC reduction (from 0.667 to 0.083 µg/mL) for tetracycline was observed against *L. monocytogenes*, at 0.5 µg/mL nitroxoline (FICI: 0.156).

The combination of tetracycline and zinc pyrithione produced synergistic antibacterial effects against five out of the six diarrhoea-causing pathogens (FICI ranging from 0.109 to 0.479; Table 2). The greatest synergistic effect was documented against *S. flexneri* (FICI value of 0.109), resulting in an 11-fold decrease in tetracycline MIC (from 5.333 to 0.5 µg/mL) at a zinc pyrithione concentration of 0.125 µg/mL. Also, the greatest tetracycline MIC reduction was reported against *V. parahaemolyticus*, at a zinc pyrithione concentration of 1 µg/mL, which resulted in a 129-fold decrease of the MIC of tetracycline (from 4 to 0.031 µg/mL) and a FICI of 0.133.

The tetracycline–sanguinarine combination demonstrated synergistic interactions against two out of the six diarrhoeic bacteria evaluated, with FICI values ranging from 0.288 to 0.5 (Table 3). The greatest synergistic effect was achieved against *L. monocytogenes* (FICI value of 0.288) at a sanguinarine concentration of 0.125 µg/mL, which caused a four-fold tetracycline MIC decrease (from 0.5 to 0.125 µg/mL). Furthermore, the greatest tetracycline MIC reduction was also observed for this bacterium (FICI of 0.428), where 1 µg/mL of sanguinarine caused an eight-fold reduction of tetracycline MIC (from 0.5 to 0.063 µg/mL).

**Figure 1:**
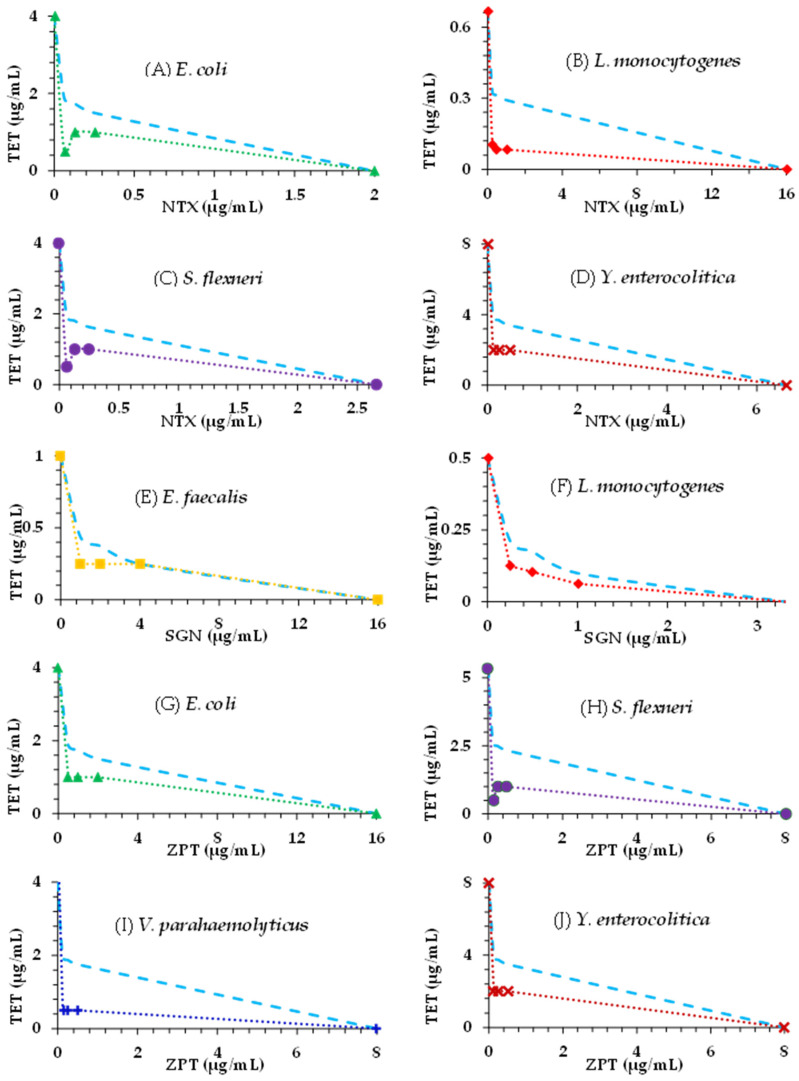
Isobolograms of the synergistic interactions for combination of tetracycline (TET) with nitroxoline (NTX) against *Escherichia coli* (**A**), *Listeria monocytogenes* (**B**), *Shigella flexneri* (**C**), and *Yersinia enterocolitica* (**D**); for combination of sanguinarine (SGN) against *Enterococcus faecalis* (**E**) and *L. monocytogenes* (**F**); and for combination of TET with zinc pyrithione (ZPT) against *E. coli* (**G**), *S. flexneri* (**H**), *Vibrio parahaemolyticus* (**I**), and *Y. enterocolitica* (**J**). *E. coli* (▲), *E. faecalis* (■), *L. monocytogenes* (♦), *S. flexneri* (●), *V. parahaemolyticus* (+), and *Y. enterocolitica* (x); border for synergy (---) calculated for ∑FIC ≤ 0.5.

Isobologram curves established from the results of the chequerboard assays and the calculated FICI values for the most susceptible bacteria are presented in Figure 1. The isobolograms confirmed the synergistic antimicrobial effects of tetracycline when combined with either nitroxoline, sanguinarine, or zinc pyrithione against *E. faecalis*, *E. coli*, *L. monocytogenes*, *S. flexneri*, *V. parahaemolyticus*, and *Y. enterocolitica*. Synergy was observed for three ratios in the isobolograms of each bacterial pathogen. The concave isobole (represented by the round dotted lines) indicates the confirmation of antimicrobial synergy observed against the tested diarrhoea-causing bacteria.

## 3. Discussion

According to the Clinical and Laboratory Standards Institute (CLSI) antimicrobial susceptibility testing breakpoint data interpretation [39], the MIC range of tetracycline observed in this study (0.5 to 8 µg/mL) proves the sensitivity of most of the diarrhoeic bacteria to this antibiotic. Previously reported MIC values for tetracycline against *E. faecalis*, *E. coli*, *V. parahaemolyticus*, and *Y. enterocolitica* were in the ranges of 0.5–32 μg/mL, 0.5–64 μg/mL, 0.06–2 μg/mL, and 2–4 μg/mL, respectively [40,41,42,43], which correspond with the findings of the current study. In contrast to the MICs determined in the current study for *L. monocytogenes* and *S. flexneri*, other researchers have reported higher MIC values (≥256 µg/mL) [44,45]. The variations in susceptibility of both bacteria to tetracycline (observed in the current study vs. the previously published data) can be explained by the use of different strains. Despite the fact that the antibacterial activities of nitroxoline, sanguinarine, and zinc pyrithione have been demonstrated in various studies, data on their in vitro growth-inhibitory effects against the diarrhoeic bacteria tested in the present study are limited. The MIC values observed for the tested antimicrobial compounds in the current study correspond well with the results of recently published data from the same laboratory, demonstrating their effectiveness against most of the tested diarrhoeic pathogens at MICs ranging from 2 to 512 µg/mL [31,46]. The synergistic effects of tetracycline and other antibiotics (e.g., amoxicillin/clavulanate and ciprofloxacin) have previously been documented for some selected diarrhoea-causing bacteria isolated from poultry droppings [47]. Although synergistic effects of nitroxoline, sanguinarine, and zinc pyrithione with antibiotics have previously been reported [31], the combined growth-inhibitory activity of these agents with tetracycline against diarrhoea-causing bacteria is reported for first time in this study. Tetracycline inhibits bacterial protein synthesis by preventing the association of aminoacyl-transfer ribonucleic acid (tRNA) with the 30S subunit of the bacterial ribosome [48]. In addition, tetracycline forms a complex with magnesium ions (Mg^2+^) and binds to the A-site of ribosomes [49,50]. Although the details of its mechanism of action are still not elucidated, it is known that the antibacterial activity of nitroxoline is due to its indirect ability to chelate cations essential for bacterial growth, especially Mg^2+^ [51,52]. Similarly, zinc pyrithione chelates metal ions, including Mg^2+^ [53]. In order to diffuse through the bacterial cell membrane, tetracycline has to be fully protonated because the Mg chelate cannot enter the cell [51]. Therefore, it is possible to assume that nitroxoline and zinc pyrithione scavenge Mg^2+^ from the environment, which creates favourable conditions for tetracycline to enter the bacterial cell membrane. Inside of the cell, tetracycline forms a complex with Mg^2+^, which is the only form which is able to inhibit bacterial growth by binding to the bacterial 30S ribosomal subunit. The chelation properties of all three agents may therefore contribute to their synergistic antibacterial activity. In the present study, the combination of sanguinarine with tetracycline produced a synergistic growth-inhibitory effect against Gram-positive bacteria. Since it has previously been suggested that the anti-staphylococcal action of sanguinarine is based on its ability to compromise the cytoplasmic membrane [54], it can be hypothesized that this compound can help tetracycline to enter the bacteria by disturbing the cell membrane. Subsequently, tetracycline can effectively inhibit bacterial protein synthesis inside the cell. Both antibacterial agents can therefore act together against *E. faecalis* and *L. monocytogenes* (as examined in the present study) through their synergistic activity.

Because both tetracycline and nitroxoline are drugs commonly used in clinical practice for the treatment of bacterial infections, their combined use could improve the efficacy of tetracycline against gastrointestinal diseases caused by diarrhoeic bacteria, because nitroxoline would chelate cations (e.g., calcium, Mg^2+^) which are reported to lower the absorption of this antibiotic in the gut [55,56]. In addition, as the above-mentioned antibacterial agents are used for the management of urinary tract infections (UTIs) [57,58], their combination could also be a potential treatment strategy against increased acquired resistance to orally administered antibiotics against UTI-causing *E. coli* [32], which has been a growing healthcare concern worldwide. Nitroxoline is an oral antibiotic used for the treatment of UTIs [59]. The use of sanguinarine in pharmacological preparations is probably not feasible because it is slightly toxic when administered orally to rats, with a median lethal dose (LD_50_) value of 1658 mg/kg [60]. According to the Scientific Committee on Consumer Safety (SCCS), zinc pyrithione is classified as a moderately toxic agent, with LD_50_ values ranging from 92 to 266 mg/kg and from 160 to 1000 mg/kg when administered orally to rats and mice, respectively [61]. Although zinc pyrithione is used as an active ingredient in hygiene and medical products, including anti-dandruff cosmetic and shampoo products [62], its application as an oral therapeutic agent seems to be limited. Nevertheless, based on the FICI values achieved in this study, the active concentrations of both sanguinarine and zinc pyrithione were greatly reduced. Therefore, when used in combination with tetracycline, lower toxicological responses in target organisms could be elicited. Further toxicological studies are needed to assess the therapeutic safety and efficacy of tetracycline in combination with the tested alkaloid-related agents before their potential pharmacological application.

## 4. Materials and Methods

### 4.1. Chemicals

Alkaloid-related agents (nitroxoline, sanguinarine chloride, and zinc pyrithione) and the antibiotic (tetracycline) used in this study were purchased from Sigma-Aldrich (Prague, Czech Republic). Dimethyl sulfoxide (DMSO) (Sigma-Aldrich, Prague, Czech Republic) was used to prepare the stock solutions of all compounds tested except tetracycline HCL, which was prepared with 96% ethanol (Penta, Prague, Czech Republic).

### 4.2. Bacterial Strains and Growth Media

The standard bacterial strains used were obtained from the American Type Culture Collection (ATCC, Rockville, MD, USA) and the National Collection of Type Cultures (NCTC, London, UK). The six different representatives of Gram-positive and -negative diarrhoea-causing bacteria that are recommended for pharmaceutical and food testing as well as for infectious and enteric disease research by the ATCC and that were assayed in this study are as follows: *E. coli* O175:H7-VT (N) NCTC 12900, *E. faecalis* ATCC 29212, *L. monocytogenes* ATCC 7644, *S. flexneri* ATCC 12022, *V. parahaemolyticus* ATCC 17802 (isolated from a case of Shirasu food poisoning in Japan), and *Y. enterocolitica* ATCC 9610. These pathogens are associated with either foodborne, waterborne, or nosocomial infections. Mueller–Hinton broth (MHB) (Oxoid, Basingstoke, UK) was used as the growth medium. This was supplemented with 3% NaCl (Sigma-Aldrich) for the culture of *V. parahaemolyticus*. All bacterial strains were grown in MHB for 24 h at 37 °C prior to testing.

### 4.3. Chequerboard Assay

In this study, a chequerboard microdilution assay was used to assess the MIC values of tetracycline and alkaloid-related agents individually and in combination, simultaneously within the same 96-well plate, following the CLSI guidelines [63] and the Clinical Microbiology Procedures Handbook [64]. For the combinations of tetracycline with each of the alkaloid-related agents, eight two-fold serial dilutions of tetracycline in the horizontal rows of the 96-well microtiter plate were cross-diluted vertically, by eight two-fold serial dilutions of the test agents, using a Freedom EVO 100 automated pipetting platform (Tecan, Mannedorf, Switzerland). The test was performed in final volume in each microplate well of 100 μL. The initial concentration for tetracycline (16 μg/mL) was further optimised depending on the susceptibility of the bacterial strains tested, whereas that of the plant-based compounds was 512 μg/mL. The inoculum was adjusted to a final bacterial concentration of 1.5 × 10^8^ CFU/mL in the MHB according to the 0.5 McFarland standard scale, using a Densi-La-Meter II (Lachema, Brno, Czech Republic). The microtiter plates were inoculated with the bacteria (5 μL/well) and incubated without agitation at 37 °C for 24 h. Afterwards, the optical density of the bacterial cultures was determined at a wavelength of 405 nm to assess growth inhibition using a Cytation 3 Imaging Reader (BioTek, Winooski, VT, USA) [65]. The MICs were expressed as the lowest concentration that inhibited bacterial growth by ≥80% compared with that of the agent-free growth control [66]. The obtained data are presented as the average values of three independent experiments, each performed in triplicate [67]. According to the widely accepted norm in MIC testing, the triplicate endpoints were always within the maximum of the three-dilution range [68], which provides (in most cases) discrete MIC values with low variability of the results. Eighteen independent sterile 96-well plates were used for the assessment of each combination of antibiotic and alkaloid-related agent (i.e., 54 plates in total); three different plates were used for each of the six different bacterial species tested. Tetracycline (tested in the same row of the microplate used for MIC determination) was also employed as a positive control for the verification of the susceptibility of the bacterial strains in the broth medium. A drug-free bacterial culture served as the negative control. There was no change in turbidity (no contamination) in the negative control wells. The highest concentration of DMSO and ethanol (both at 1%) present in the microtiter plates did not inhibit the bacterial growth of any strain tested.

### 4.4. Evaluation of Combination Effects

The FICI (=∑FIC), a measure recommended by the European Committee on Antimicrobial Susceptibility Testing, has been used for the assessment of combinatory effect of antibacterial agents [69]. The combined effects of tetracycline (A) and alkaloid-related agents (B) were calculated using the following equation: ∑FIC = FIC_A_ + FIC_B_ where, FIC_A_ = MIC_A combination B_/MIC_A alone_ and FIC_B_ = MIC_B combination A_/MIC_B alone_ [70]. With the aim of avoiding reproducibility errors in MIC chequerboard interpretation, the effects were evaluated according to strict criteria proposed by Odds [71] using average values of the FICIs. The results were interpreted as follows: synergy if ∑FIC ≤ 0.5; no interaction if ∑FIC > 0.5–4; and antagonism if ∑FIC > 4. For the purpose of describing the synergistic interactions of the antimicrobial agents, the minimum FICI values were used. To aid in the interpretation of the results in Table 1, Table 2 and Table 3, graphical representations of the FICI data obtained for the most sensitive bacteria are illustrated in the form of isobolograms (Figure 1). Following the description, a synergy effect is shown by an upward concave isobole, no interaction is shown by a straight line on the *x* and *y*-axis (linear isobole), and an antagonistic effect is represented by a convex isobole [72]. The axes of each isobologram represent the dose-axes of the individual agents. The border of synergy was calculated according to the equation 0.5 − (MIC_A combination B_/MIC_A alone_) × MIC_B alone_, which is based on the conservative interpretation of results eliminating reproducibility errors in MIC values determined by the chequerboard methodology [71]. The standard deviation, percentage coefficient of variation, and probability of synergy success (PSS; PSS = number of synergistic interactions/total number of experiments {*n* = 3}) of the mean data (Table 1, Table 2 and Table 3) are presented in the Supplementary Materials (Tables S1 and S2, etc.). The synergistic effect occurred when PSS value is 100%. Microsoft Excel 365 (Microsoft, Redmond, WA, USA) was used for statistical data analysis.

## 5. Conclusions

In this study, tetracycline in combination with either nitroxoline, sanguinarine, or zinc pyrithione produced antibacterial synergistic interactions against most of the diarrhoea-causing bacteria tested. The best result was obtained when tetracycline and nitroxoline were used against *S. flexneri*, resulting in a FICI value of 0.086. To the best of our knowledge, this is the first report of the synergistic interactions between tetracycline and the above-mentioned alkaloid-related compounds against most of the selected diarrhoeic genic bacteria strains tested. In addition, based on the FICI values obtained in this study, the synergistic actions suggested that the combination of the antimicrobials was more active against the diarrhoeic microorganisms than the activity of the single agents alone. Furthermore, because of its ability to potentiate the growth-inhibitory effect of tetracycline against *E. faecalis*, *E. coli*, *L. monocytogenes*, *S. flexneri*, *V. parahaemolyticus*, and *Y. enterocolitica* at a decreased concentration, nitroxoline seems to be an attractive candidate for the development of new synergistically acting anti-diarrhoeic medications for humans. Nevertheless, further research that focuses on their in vivo anti-diarrhoeic activity and safety is needed before any consideration for use in human medicine.

## Figures and Tables

**Table 1:** In vitro susceptibility of diarrhoeic bacteria to tetracycline (TET) and nitroxoline (NTX) alone and in combination.

Bacterium	MIC ^a^ Alone		NTX MIC (in Bold)											
	TET	NTX	8		4		2		1		0.5		0.25	
			TET MIC	FIC I ^b^	TET MIC	FICI	TET MIC	FICI	TET MIC	FICI	TET MIC	FICI	TET MIC	FICI
*E. faecalis*	1	16	0.031	0.531	0.25	0.5	0.25	0.375	0.25	0.313	1	1.031	1	1.016
*L. monocytogenes*	0.667	16	0.052	0.578	0.109	0.413	0.083	0.249	0.083	0.187	0.083	0.156	0.104	0.172
			**2**		**1**		**0.5**		**0.25**		**0.125**		**0.063**	
*E. coli* O175:H7	4	2	0.063	1.016	1	0.75	4	1.25	1	0.375	1	0.313	0.5	0.157
*S. flexneri*	4	2.667	0.063	0.766	2.333	0.958	3	0.937	1	0.344	1	0.297	0.25	0.086
*V. parahaemolyticus*	2	2	0.016	1.008	0.016	0.508	0.5	0.50	0.5	0.375	0.5	0.313	0.5	0.282
			**1**		**0.5**		**0.25**		**0.125**		**0.063**		**0.031**	
*Y. enterocolitica*	8	6.667	2	0.400	2	0.325	2	0.287	2	0.269	2	0.259	2	0.255

^a^ MIC, minimum inhibitory concentration of TET and NTX expressed as an average of three independent experiments, each performed in triplicate. All MICs units are in µg/mL. ^b^ FICI, fractional inhibitory concentration index; FICI values (≤0.5) indicate synergistic effects; FICI values (>0.5–4) indicate no interaction effect; FICI values (>4) indicate antagonistic effect.

**Table 2:** In vitro susceptibility of diarrhoeic bacteria to tetracycline (TET) and zinc pyrithione (ZPT) alone and in combination.

Bacterium	MIC ^a^ Alone		ZPT MIC (in Bold)											
	TET	ZPT	4		2		1		0.5		0.25		0.125	
			TET MIC	FICI ^b^	TET MIC	FICI	TET MIC	FICI	TET MIC	FICI	TET MIC	FICI	TET MIC	FICI
*S. flexneri*	5.333	8	1	0.688	1	0.438	1	0.313	1	0.250	1	0.219	0.5	0.109
*V. parahaemolyticus*	4	8	0.016	0.504	0.031	0.258	0.031	0.133	0.5	0.188	0.5	0.156	0.5	0.141
			**2**		**1**		**0.5**		**0.25**		**0.125**		**0.063**	
*E. coli* O175:H7	4	16	1	0.375	1	0.313	1	0.281	2	0.516	2	0.508	1	0.254
*E. faecalis*	1	8	0.5	0.75	1	1.125	1	1.063	1	1.031	1	1.016	1	1.008
*Y. enterocolitica*	8	8	1	0.375	1	0.25	2	0.313	2	0.281	2	0.266	2	0.258
			**1**		**0.5**		**0.25**		**0.125**		**0.063**		**0.031**	
*L. monocytogenes*	0.5	8	0.125	0.375	0.208	0.479	0.208	0.447	0.125	0.266	0.125	0.258	0.125	0.254

^a^ MIC, minimum inhibitory concentration of TET and ZPT expressed as an average of three independent experiments, each performed in triplicate. All MICs units are in µg/mL. ^b^ FICI, fractional inhibitory concentration index; FICI values (≤0.5) indicate synergistic effects; FICI values (>0.5–4) indicate no interaction effect; FICI values (>4) indicate antagonistic effect.

**Table 3:** In vitro susceptibility of diarrhoeic bacteria to tetracycline (TET) and sanguinarine (SGN) alone and in combination.

Bacterium	MIC ^a^ Alone		SGN MIC (in Bold)											
	TET	SGN	16		8		4		2		1		0.5	
			TET MIC	FICI ^b^	TET MIC	FICI	TET MIC	FICI	TET MIC	FICI	TET MIC	FICI	TET MIC	FICI
*E. coli* O175:H7	4	128	4	1.125	4	1.063	4	1.031	4	1.016	2	0.508	2	0.504
			**8**		**4**		**2**		**1**		**0.5**		**0.25**	
*E. faecalis*	1	16	0.031	0.531	0.25	0.5	0.25	0.375	0.25	0.313	1	1.031	1	1.016
*Y. enterocolitica*	2	64	2	1.125	2	1.063	2	1.031	2	1.016	2	1.008	2	1.004
			**4**		**2**		**1**		**0.5**		**0.25**		**0.125**	
*L. monocytogenes*	0.5	3.333	0.016	1.232	0.031	0.662	0.063	0.458	0.104	0.358	0.125	0.325	0.125	0.288
			**2**		**1**		**0.5**		**0.25**		**0.125**		**0.063**	
*S. flexneri*	1	16	1	1.125	1	1.063	0.5	0.531	0.5	0.516	1	1.008	1	1.004
*V. parahaemolyticus*	1	16	0.5	0.625	0.5	0.563	1	1.031	1	1.016	1	1.008	1	1.004

^a^ MIC, minimum inhibitory concentration of TET and SGN expressed as an average of three independent experiments, each performed in triplicate. All MICs units are in µg/mL. ^b^ FICI, fractional inhibitory concentration index; FICI values (≤0.5) indicate synergistic effects; FICI values (>0.5–4) indicate no interaction effect; FICI values (>4) indicate antagonistic effect.

## Data Availability

The original contributions presented in this study are incorporated in the article/Supplementary Materials, and further inquiries can be directed to the corresponding author.

## Supplementary Materials

The following supporting information can be downloaded at: https://www.mdpi.com/article/10.3390/ijms25116038/s1.
