# Supplementary material for: Evaluation of In Vitro Synergistic Effects of Tetracycline with Alkaloid-Related Compounds against Diarrhoeic Bacteria
Source: Int J Mol Sci. 2024 May 30;25(11):6038. doi: 10.3390/ijms25116038 (PMC11173066; doi:10.3390/ijms25116038)
Supplement: Supplementary file 1 [file ijms-25-06038-s001.zip › ijms-3021790-supplementary.pdf]

International Journal of *Molecular Sciences*

Article

Evaluation of *in vitro* synergistic effects of tetracycline with alkaloid-related compounds against diarrhoeic bacteria

Hayford Osei-Owusu[1], Johana Rondevaldova[1], Marketa Houdkova[1], Tomas Kudera[1], Tersia Needham[2], Anna Mascellani[3] and Ladislav Kokoska[1]*

1 Department of Crop Sciences and Agroforestry, Faculty of Tropical AgriSciences, Czech University of Life Sciences Prague, Kamycka 129, 165 00 Prague - Suchdol, Czech Republic, Hayford Osei-Owusu Osei-Owusu@ftz.czu.cz, Johana Rondevaldova rondevaldova@ftz.czu.cz, Marketa Houdkova houdkovam@ftz.czu.cz, Tomas Kudera kuderat@ftz.czu.cz, Ladislav Kokoska* kokoska@ftz.czu.cz (*corresponding author).

2 Department of Animal Science and Food Processing, Faculty of Tropical AgriSciences, Czech University of Life Sciences Prague, Kamycka 129, 165 00 Prague - Suchdol, Czech Republic, Tersia Needham needham@ftz.czu.cz.

3 Department of Food Science, Faculty of Agrobiology, Food and Natural Resources, Czech University of Life Sciences Prague, Kamycka 129, 165 00 Prague Suchdol, Czech Republic, Anna Mascellani mascellani@af.czu.cz

Citation: To be added by editorial staff during production.

Academic Editor: First name Lastname

Received: date
Revised: date
Accepted: date
Published: 30 May 2024

Copyright: © 2024 by the authors. Licensee MDPI, Basel, Switzerland. This article is an open access article distributed under the terms and conditions of the Creative Commons Attribution (CC BY) license (https://creativecommons.org/licenses/by/4.0/).

Supplementary material: Supplementary figure

Table S1. Graph showing a layout of experiment for chequerboard display with automatically generated for tetracycline in combination with nitroxoline against *Escherichia coli.*

	S	Q								X	Y	Z
A	Sterility control	8/8	4/8	2/8	1/8	0.5/8	0.25/8	0.125/8	0.0625/8	16	16	Growth control
B		8/4	4/4	2/4	1/4	0.5/4	0.25/4	0.125/4	0.0625/4	8	8	
C		8/2	4/2	2/2	1/2	0.5/2	0.25/2	0.125/2	0.0625/2	4	4	
D		8/1	4/1	2/1	1/1	0.5/1	0.25/1	0.125/1	0.0625/1	2	2	
E		8/0.5	4/0.5	2/0.5	1/0.5	0.5/0.5	0.25/0.5	0.125/0.5	0.0625/0.5	1	1	
F		8/0.25	4/0.25	2/0.25	1/0.25	0.5/0.25	0.25/0.25	0.125/0.25	0.0625/0.25	0.5	0.5	
G		8/0.125	4/0.125	2/0.125	1/0.125	0.5/0.125	0.25/0.125	0.125/0.125	0.0625/0.125	0.25	0.25	
H		8/0.0625	4/0.0625	2/0.2065	1/0.0625	0.5/0.0625	0.25/0.0625	0.125/0.0625	0.0625/0.0625	0.125	0.125	

Table S2. a) Layout of experiment showing concentrations (two-fold serial dilution) of tested agents in combination (Q), X: Compound A (antibiotic) alone in two-fold dilutions (starting at concentration 16 µg/mL); Y: Compound B (alkaloid-related agent) in two-fold dilutions (starting at concentration 16 µg/mL); S: Sterility control (non-infected medium control; 0% growth of bacterium); Z: Growth control (infected medium control; 100% growth of bacterium). b) Values of optical density readings of microtiter plate at wavelength 405 nm for combination of tetracycline with nitroxoline against *Escherichia coli*.

	1	2	3	4	5	6	7	8	9	10	11	12
A	0.115	0.22	0.22	0.215	0.231	0.224	0.236	0.218	0.226	0.201	0.291	1.136
B	0.196	0.345	0.291	0.291	0.287	0.295	0.304	0.308	0.296	0.275	0.322	1.519
C	0.211	0.286	0.283	0.273	0.266	0.268	0.281	0.29	0.262	0.377	0.3	1.422
D	0.227	0.27	0.303	0.37	0.386	0.327	0.408	0.825	1.005	0.583	0.365	1.58

E	0.129	0.27	0.309	0.324	0.342	0.445	0.458	1.455	1.478	0.798	1.428	1.469
F	0.33	0.266	0.391	0.333	0.371	0.467	1.491	1.294	1.439	1.194	1.569	1.508
G	0.211	0.257	0.341	0.349	0.325	0.459	1.395	1.435	1.45	1.357	1.596	1.422
H	0.194	0.203	0.215	0.321	0.358	0.421	1.303	1.22	1.37	1.311	1.388	1.271

Disclaimer/Publisher's Note: The statements, opinions and data contained in all publications are solely those of the individual author(s) and contributor(s) and not of MDPI and/or the editor(s). MDPI and/or the editor(s) disclaim responsibility for any injury to people or property resulting from any ideas, methods, instructions or products referred to in the content.
